# All Roads Lead to the Gut: The Importance of the Microbiota and Diet in Migraine

**DOI:** 10.3390/neurolint15030073

**Published:** 2023-09-13

**Authors:** Eleonóra Spekker, Gábor Nagy-Grócz

**Affiliations:** 1 Pharmacoidea Ltd., H-6726 Szeged, Hungary; 2 Department of Neurology, Albert Szent-Györgyi Medical School, University of Szeged, H-6725 Szeged, Hungary; nagy-grocz.gabor@szte.hu; 3 Faculty of Health Sciences and Social Studies, University of Szeged, H-6726 Szeged, Hungary; 4 Preventive Health Sciences Research Group, Incubation Competence Centre of the Centre of Excellence for Interdisciplinary Research, Development and Innovation of the University of Szeged, H-6720 Szeged, Hungary

**Keywords:** migraine, headache, gut microbiome, gut-bran axis, nutrition, dietary triggers, diets, prebiotics, probiotics

## Abstract

Migraine, a prevalent neurological condition and the third most common disease globally, places a significant economic burden on society. Despite extensive research efforts, the precise underlying mechanism of the disease remains incompletely comprehended. Nevertheless, it is established that the activation and sensitization of the trigeminal system are crucial during migraine attacks, and specific substances have been recognized for their distinct involvement in the pathomechanism of migraine. Recently, an expanding body of data indicates that migraine attacks can be prevented and treated through dietary means. It is important to highlight that the various diets available pose risks for patients without professional guidance. This comprehensive overview explores the connection between migraine, the gut microbiome, and gastrointestinal disorders. It provides insight into migraine-triggering foods, and discusses potential diets to help reduce the frequency and severity of migraine attacks. Additionally, it delves into the benefits of using pre- and probiotics as adjunctive therapy in migraine treatment.

## 1. Migraine

Migraine ranks among the most prevalent neurological conditions, is a major cause of socio-economic and health problems worldwide, and affects approximately 12% of the population [1,2,3]. Repeated migraine attacks can make sufferers physically, mentally, and socially incapacitated for several days [4].

**Figure 1:**
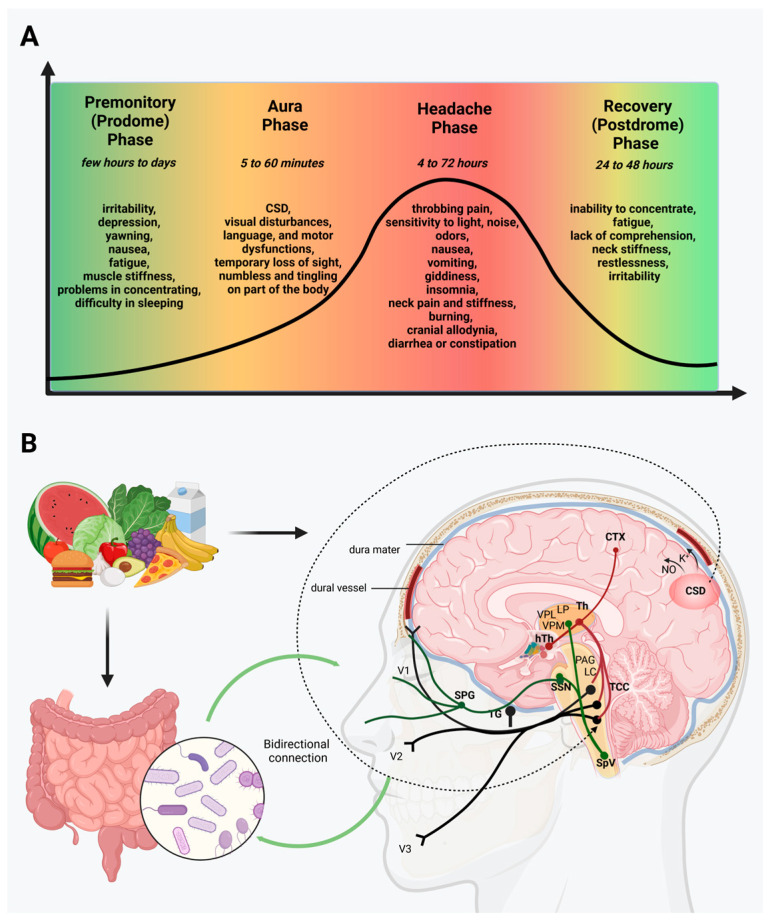
Migraine’s phases and the factors assumed to be involved in its pathomechanism. (**A**) The four phases of migraine: the premonitory (prodrome) phase, a possible aura, the headache, and recovery (post-drome). (**B**) Vascular dysfunction, CSD, activation of the trigeminovascular pathway, and inflammatory and oxidative conditions may play a fundamental role in the development of mi-graine pain. Moreover, nutrition and the composition and function of the gut microbiome influence the development of migraine attacks. The trigeminal ganglion (TG) originates pseudo-unipolar trigeminal primary sensory neurons that establish connections with both intra- and extracranial structures, including blood vessels, as well as the spinal cord’s trigeminocervical complex (TCC) (black line). Second-order neurons arising from the TCC ascend via the trigeminothalamic tract, where they form synapses with third-order thalamocortical neurons. There are also direct and indirect ascending projections to the locus coeruleus (LC), periaqueductal gray (PAG), and hypothalamus. Subsequently, these third-order thalamocortical neurons synapse within an extensive network of cortical regions (red line). There is also activation of the parasympathetic reflex through the outflow from the superior salivatory nucleus (SSN) via the facial nerve, predominantly involving the sphenopalatine ganglion (SPG), which acts to dilate blood vessels and activate trigeminal nerve endings (green line). CTX, cortex; NO, nitric oxide; CSD, cortical spreading depression; Th, thalamus; hTh, hypothalamus; LP, lateral posterior nucleus; VPM, ventral posteromedial nucleus; VPL, ventral posterolateral nucleus; PAG, periaqueductal grey matter; LC, locus coeruleus; TCC, trigeminocervical complex; SSN, superior salivatory nucleus; SpV, spinal trigeminal nucleus caudalis; TG, trigeminal ganglion; SPG, sphenopalatine ganglion; V1, ophthalmic nerve; V2, maxillary nerve; V3, mandibular nerve.

Four distinct phases of the clinical course of migraine have been identified: the premonitory (prodrome) phase, a possible aura, the headache, and recovery (postdrome) phase, and their occurrence is not necessarily linear [2]. The premonitory phase occurs hours or days before the headache and is characterized by, among other things, irritability, fatigue, concentration difficulties, neck stiffness [5]. Behavioral changes that affect mood, appetite, and energy indicate the involvement of the hypothalamus [6]. This belief is substantiated by the participation of several hypothalamic neurotransmitters in migraine, including orexin, dopamine, somatostatin, melatonin, cholecystokinin, and antidiuretic hormone [7,8]. In 25% of migraine sufferers, the aura occurs, which is a reversible neurological phenomenon. It most often manifests itself in the form of visual disturbances, but it can also cause other sensory, language, and motor dysfunctions [2,9]. The clinical occurrence known as migraine aura is thought to be a temporary wave of depolarization among cortical neurons, referred to as cortical spreading depression (CSD) [5]. The headache phase is characterized by attacks of a moderate-to-severe unilateral headache (lasting for 4–72 h) accompanied by a variety of other symptoms, such as sensitivity to light, sounds, and odors, cranial allodynia, and some gastrointestinal (GI) symptoms including nausea, vomiting, diarrhea, or constipation. Furthermore, physical activity can contribute to worsening pain [1,2,3]. The postdrome phase is the last stage of a migraine attack, and can include many symptoms such as neck stiffness, difficulty concentrating, fatigue, restlessness, and irritability [10] (Figure 1).

Despite intensive research, the pathogenesis of migraine disease remains poorly understood. At the same time, vascular dysfunction, CSD, activation of the trigeminovascular pathway, and inflammatory and oxidative conditions may play a fundamental role in the development of migraine pain [11,12,13,14,15]. During the activation of the trigeminal system, neurotransmitters, such as calcitonin gene-related peptide (CGRP), substance P (SP), pituitary adenylate cyclase-activating polypeptide (PACAP), and neurokinin A (NKA), are released from the primary sensory neurons and induce mast cell degranulation and plasma extravasation, which can eventually lead to the development of neurogenic inflammation [14,16].

Numerous endogenous (such as gene variants and hormones) and exogenous (such as diet and drugs) factors contribute to the severity and frequency of migraine [17,18]. The most common migraine triggers are stress, fatigue, fasting, lack of sleep, and the weather. In addition, about 20% of migraine sufferers report food as a migraine trigger [19,20].

In the gut, inflammatory mediators act as sensitizers of afferent endings. In addition, pro-inflammatory cytokines such as interleukin (IL)-1β, IL-6, IL-8, and tumor necrosis factor-α (TNF-α) are increased during migraine attacks [21,22]. Furthermore, CGRP, SP, vasoactive intestinal peptide (VIP), and neuropeptide Y (NPY), are thought to have an antimicrobial impact on a variety of gut bacterial strains; thus, they can be involved in the bidirectional gut–brain communication [23]. Another fundamental factor contributing to this relationship is altered serotonergic signaling, which can activate the trigeminovascular system and lead to the development of gastric symptoms, including nausea, vomiting, and delayed gastric emptying, which occur in both GI disorders and migraine [24,25]. A growing number of GI disorders are associated with migraines, suggesting that gut microbiota may play a key role in this disease [26]. Moreover, several recently published studies have suggested that diet plays a significant role in migraine, so dietary changes may be useful in headache prevention and treatment [18,25,27] (Figure 1).

Although the number of studies on the effects of gut microbiota composition and diet on migraine is not yet large, the present review summarizes the available evidence.

## 2. Gut Microbiota and Brain

The gut microbiota consists of bacteria, viruses, protozoa, and fungi present in the GI tract. These microorganisms have a tremendous impact on our physiology, both in health and in disease; among other things, they contribute to metabolic functions, influence brain–gut communication, provide protection against pathogens, and affect the immune system [28]. The human metabolism greatly benefits from the involvement of the gut microbiome in producing enzymes, as well as synthesizing essential vitamins like biotin, thiamine, cobalamin, riboflavin, nicotine, and pantothenic acids, along with branched-chain amino acids, phenols, and indoles [29]. In addition, they can metabolize indigestible carbohydrates such as cellulose, starch, and pectin into short-chain fatty acids (SCFAs) [30].

**Figure 2:**
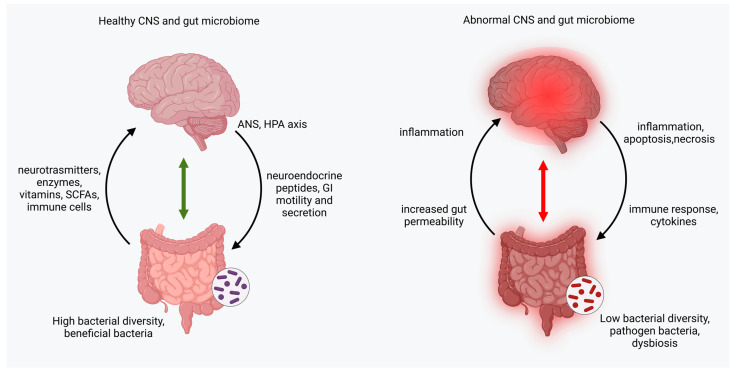
Bidirectional connection between the brain and the gut microbiome in both a healthy and abnormal state. CNS, central nervous system; ANS, autonomic nervous system; HPA, hypothalamic–pituitary–adrenal; SCFAs, short-chain fatty acids; GI, gastrointestinal.

Numerous studies have demonstrated that there is a complex and diverse interaction between the gut microbiome and the central nervous system (CNS). This specific connection is known as the “gut–brain axis”, a bidirectional relationship between the two, which includes afferent and efferent neural, endocrine, nutrient, and immunological signals [31] (Figure 2). The gut–brain axis serves as a coordination system for gut functions and maintenance, linking the emotional centers of the brain with peripheral intestinal mechanisms, including enteric reflexes, intestinal permeability, immune responses, and entero-endocrine signaling [32].

The composition of the gut microbiota plays an important role in the gut–brain axis. Gut microbiota can affect the CNS through two mechanisms: microbiota-derived neurotransmitters, inflammatory molecules, and hormones; and through a direct connection with the stimulating end terminals of the vagus nerve [25]. At the same time, the CNS can modulate the gut microbiota through the sympathetic and parasympathetic systems and the release of neuroendocrine peptides [23] (Figure 2).

Several diseases are now thought to be influenced by processes in the gut microbiome. Those include cancer, autoimmune disorders, cardiovascular diseases, and various neurological and psychiatric disorders [33]. The gut–brain axis has shown signs of dysfunction in various neurological disorders, including multiple sclerosis, Alzheimer’s disease, and migraine [34] (Figure 2).

As the gut microbiota plays a crucial role in host health, manipulation of the gut microbiome, such as diets and pre- and probiotic supplementation to restore the balance of disturbed gut microbiota, may offer opportunities for disease prevention and mitigation [35,36].

## 3. Migraine, the Microbiome, and GI Disorders

The gut–brain axis can trigger a migraine attack in many ways, e.g., through the composition of the gut microbiome, neuropeptides, stress hormones and nutrients. Different stressors (physical or psychological) can lead to dysbiosis (an imbalance or disruption in the composition and function of the microbial communities), which causes an increase in the secretion of CGRP, which correlates with the symptoms observed during migraine attacks [25,37]. In addition, increased secretion of serum cytokines (ILs, TNF-α)—an important regulator of inflammatory responsiveness—has been observed during migraine attacks [38].

Many neurotransmitters are involved in pain perception; the most specific of these are glutamate and gamma-aminobutyric acid (GABA), which are widely distributed in the body [39]. These neurotransmitters in the gut are involved in several signaling pathways that, in addition to modulating pain, regulate the release of pro-inflammatory cytokines [40]. Research has verified that various bacterial strains, including those found in the environment or employed in food fermentation, are capable of generating glutamate [41]. Since glutamate can exert a stimulating effect on nociceptive neurons along the trigeminovascular pathway, it may be crucial in the pathophysiology of migraine headaches and migraine-related central sensitization. This theory is further proven by the fact that elevated blood levels of glutamate have been recorded in migraine patients both interictally and ictally. In addition, there is evidence that glutamate plays a major role in CSD, which is hypothesized to be the physiological substrate of migraine aura [42]. Based on these, glutamatergic neurotransmission may be a link between migraine and the microbiome.

An imbalance in the gut microbiota has been demonstrated to play a role in the development of migraine [40]. Nitrates have been identified as a prevalent migraine trigger. Higher levels of bacterial species capable of reducing nitrates, nitrites, and nitric oxide, such as Haemophilus sp. and Rothia sp., were observed in the oral and fecal samples of individuals with migraines compared to those without the condition [43]. It was also observed that in migraineurs, the species diversity and metabolic functions of gut microbiota decreased, and the number of Clostridium species (e.g., *Cl. asparagiforme*, *Cl. clostridioforme*, *Cl. bolteae*, *Cl. citroniae*, *Cl. hathewayi*, *Cl. ramosum*, *Cl. spiroforme*, *Cl. symbiosum*) increased, as opposed to beneficial species, which is more common in non-migraine subjects [44].

**Figure 3:**
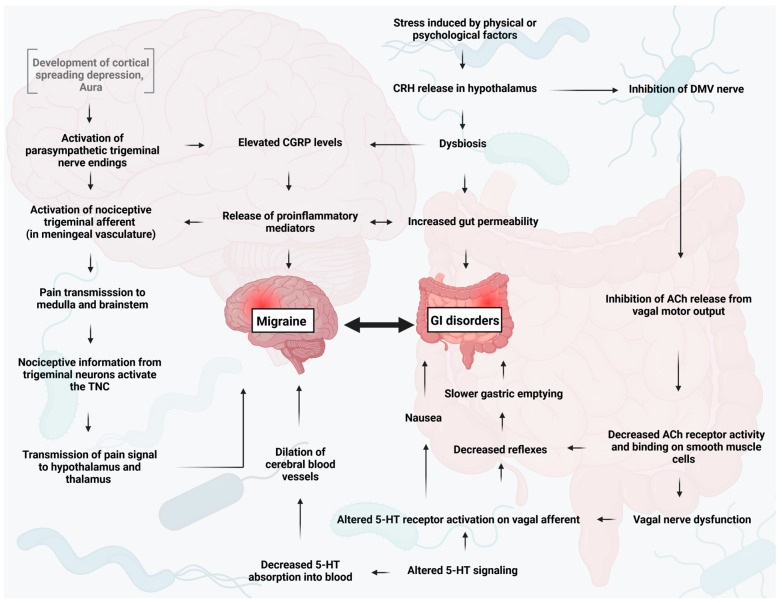
The relationship between migraine, gastrointestinal disorders and microbiota. Changes in sympathetic and parasympathetic activity and the gut microbiota profile—mediated by different cytokines, hormones and neurotransmitters—contribute to the development of migraine and GI diseases [46]. CGRP, calcitonin gene-related peptide; CRH, corticotrophin-releasing hormone; ACh, acetylcholine; 5-HT, serotonin; TNC, trigeminal nucleus caudalis; DMV, dorsal motor nucleus of the vagus; GI; gastrointestinal.

Since the microbiome contributes to other neurological disorders, it is not surprising that a connection between migraine and gastrointestinal (GI) disorders has been noted, with the frequency of GI complaints rising in tandem with the frequency of headaches [45] (Figure 3). There is evidence that migraine and GI disorders share a common pathophysiology, which is thought to occur through the interaction of several factors, including inflammatory mediators, gut microbiota, neuropeptides, and the serotonin (5-HT) pathway [25,46].

An increased inflammatory immune response has been observed in both inflammatory diseases and migraines. During migraine attacks, increased levels of pro-inflammatory cytokines such as TNF-α and IL-1β have been detected in the serum [47,48]. The main trigger of pro-inflammatory immune responses is the entry of lipopolysaccharides (LPS) into the circulation as a result of increased gut permeability. Consequently, inflammatory responses may manifest in various body regions, such as the activation of nociceptors on the trigeminal nerve in the context of migraine [48].

Furthermore, an increasing body of research indicates a heightened occurrence of migraines in individuals diagnosed with irritable bowel syndrome (IBS) and inflammatory bowel disease (IBD) [49,50,51]. Both are severe intestinal diseases associated with increased gut permeability and inflammation caused by microbes. Moreover, central, visceral, and thermal cutaneous hypersensitization are common among IBS and migraine [49]. Migraine patients with long-standing and more frequent headaches were more likely to be diagnosed with IBS [52].

In gastroparesis, gastric emptying time was observed to be significantly correlated with the severity of headache, nausea, and sensitivity to light in patients with migraine attacks [53]. Domperidone, a dopamine receptor antagonist, can be used to treat gastroparesis and has been shown to prevent most migraine attacks when administered early in higher doses [54,55]. Another dopamine receptor antagonist, metoclopramide, can be used to treat gastroparesis and nausea, and is effective as an acute intravenous treatment for migraine [56,57].

Individuals diagnosed with celiac disease (CD), an autoimmune disorder triggered by gluten peptides, exhibit a higher occurrence of migraines in comparison to those without the condition, and conversely, individuals with migraines have a higher prevalence of CD [50,58,59]. The link between migraines and CD can be attributed to several simultaneous mechanisms, encompassing the activation of pro-inflammatory cytokines due to gluten, deficiencies in vital vitamins and essential elements, disturbances in vascular tone, and an increased sensitivity of the nervous system [26,60].

Unfortunately, the exact mechanisms behind the involvement of the gut microbiota in migraine are unknown.

## 4. Migraine and Diets

### 4.1. Nutrition and Dietary Triggers

Nutrition and dietary triggers may be an important factor in migraine prevention, since we know that migraine attacks can be triggered by certain dietary compounds [18]. It is well known that in the treatment of several disorders, e.g., obesity [61] and metabolic syndrome [62], personalized or precision nutrition (PN) is used. PN establishes dynamic and comprehensive nutritional guidance based on personal differences, including genetics, metabolic profile, microbiome, physical activity, health status, food environment, dietary pattern, and socioeconomic and psychosocial features. The pathomechanism of migraine can be linked to metabolic endocrine disorders and metabolic processes [63,64], so PN may work as a promising supplementary therapy for migraine in the future.

#### Potential Dietary Triggers in Migraine

**Figure 4:**
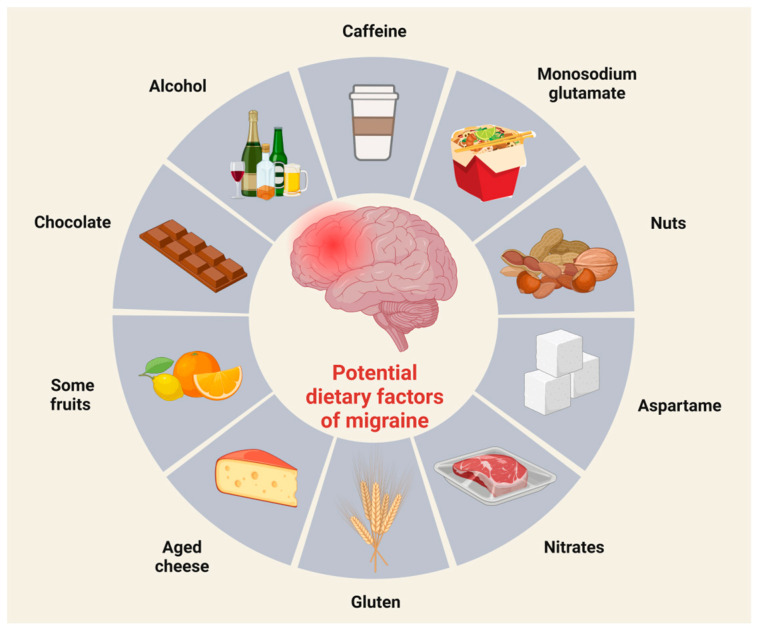
Potential dietary factors of migraine. Scientific evidence suggests that certain foods can trigger migraine attacks. Common migraine-triggering foods include chocolate, coffee, and red wine.

Many dietary compounds are known as potential migraine triggers, which can influence the frequency of attacks depending on the individual (Figure 4) [65,66].

The reaction of patients to these above-mentioned ingredients depends on genetic factors, quantity, and time of exposure [65], with most experiment findings concerning monosodium glutamate, caffeine, and alcohol.

Monosodium glutamate is a flavor enhancer and can be found in canned and frozen foods, salad dressings, soups, snack foods, ketchup, and barbecue sauces [67]. Based on literature data, monosodium glutamate might be a migraine trigger in high concentrations and dissolved in liquids, but not as a component of solid foods [68], which proves that it is not possible to generalize and say that one ingredient can provoke migraine attacks in every form and in every patient.

The other often-mentioned compound in association with migraine is caffeine, known for its ability to eliminate or provoke attacks. In combination with aspirin and acetaminophen, caffeine is a highly efficient pain killer. On the other hand, it is well known that caffeine withdrawal can initiate migraine attacks in caffeine users [69]. Regarding caffeine, people must pay attention to their dosage, as we know that low amounts of caffeine (~200 mg per day) have no unsafe effects contribute to decreasing the symptoms of depression [70]. Based on the literature data, we have inconclusive results concerning the connection between caffeine and migraine frequency. Some research data show that different headaches, migraine, and chronic daily headaches are more common in caffeine users than in people who do not consume this substance [71]. In contrast, other observations indicate that there is no association between headache, migraine frequency, and caffeine consumption [72].

Another substance that is the focus of attention when it comes to migraine prevention is alcohol. Ethanol can stimulate meningeal nociceptors in the trigeminal ganglion, triggering pain signals that are then transmitted in the spinal trigeminal nucleus to the thalamic nuclei, and finally to the somatosensory cortex [73]. Other mechanisms may also be involved, e.g., the vasodilator effect, dehydration, toxicity, etc. [74]. In addition to alcohol, alcoholic beverages also contain certain compounds (the byproducts of alcohol fermentation) that can trigger migraine attacks [73]. Hindiyeh and her colleagues have shown in an exceptional systematic review that alcohol consumption is one of the most common causes of migraine attacks, and the most frequently mentioned alcoholic drink is red wine [27].

Numerous studies have proposed an association between chocolate consumption and headaches, yet the precise physiological mechanisms responsible remain unclear [20,75]. One possible explanation for why chocolate triggers migraine attacks may be that the flavanols in it stimulate endothelial nitric oxide synthase (eNOS) activity, which can lead to vasodilation through increased nitric oxide (NO) production [76]. However, the results are contradictory [77,78]. Another possible cause is 5-HT. It is well known, that the concentration of 5-HT increases during a migraine attack. Moreover, 5-HT and its precursor tryptophan were found in chocolate. It is possible that by increasing 5-HT levels, chocolate consumption can trigger a migraine attack. However, there are many studies that support the beneficial effects of chocolate [78,79,80,81]. Chocolate contains many vitamins and minerals (for example, magnesium and riboflavin) that are recommended for migraine prevention [81]. Furthermore, Cady and colleagues discovered that a diet enriched with cocoa prevented inflammatory reactions in trigeminal ganglion neurons by suppressing the expression of CGRP [77].

Tyramine, an amine compound derived from the amino acid tyrosine, is present in various food items, including aged cheeses, cured meats, smoked fish, beer, fermented foods, and yeast extract, among others [82]. Tyramine has the potential to trigger headaches by promoting the release of norepinephrine and exerting an agonistic influence on α-adrenergic receptors [83].

Aspartame is an artificial sweetener. Several studies suggest that it causes various neurological or behavioral symptoms; in addition, its use can cause headaches, especially in people who consume moderate or high doses (900–3000 mg/day) for a long time [84,85,86].

Based on the above, recognizing and avoiding dietary migraine triggers is essential, as it can help reduce the frequency of migraines, allowing migraineurs to gain control over a condition that leaves them feeling exhausted and helpless.

### 4.2. Diets

#### 4.2.1. Elimination Diets

As we discussed earlier, patients are also different in what compounds can trigger an attack in them. Based on this fact, they can avoid ingredients that have provoked migraine for them already. This process is called an elimination diet. Randomized crossover studies and double-blind, randomized, crossover trials have found that elimination diets can reduce attack frequency, duration, severity, and the amount of medication required to counter these attacks [87,88]. Using this method is not guaranteed to resolve migraines, because migraine attacks are almost always multi-triggered; thus, eliminating or identifying one compound is not a failsafe way to avoid migraine. Electronic diaries and artificial intelligence might help us use big data from patients and identify headache-associated ingredients [18,87].

#### 4.2.2. Migraine and Other Targeted Diets

Researchers have developed targeted migraine diets [18] that influence many “migraine-specific” areas and mechanisms of the body, namely brain mitochondrial function, neuroinflammation, NO, CGRP, or neuronal excitability [89].

Among these diets, the ketogenic diet has increasingly come into focus in the treatment of neurological disorders, including migraine, which balances severe restriction of carbohydrates with higher intakes of lipids and proteins. Ketogenic diets increase the number of ketone bodies, which has a beneficial effect on migraine prevention. Ketogenic diets and ketone bodies have roles in enhancing neuroprotection, acting against serotonergic dysfunctions, repairing mitochondrial function, suppressing neuroinflammation, and reducing CGRP levels in patients [89]. Thus, enhancing ketone bodies might have a positive effect on migraine prevention [90]. In a 2016 study, adherence to the ketogenic diet for 1 month was found to be able to decrease the frequency and duration of migraine attacks in a small group of patients [91].

Besides ketogenic diets, patients should also know about the benefits of consuming omega-3 and omega-6 fatty acids. Balancing these acids is not only important in migraine prevention and treatment, but it is also crucial in preventing other disorders, e.g., atherosclerosis, as well. Decreasing omega-6 and enhancing omega-3 fatty acids in the body might have a positive effect in trying to reduce migraine attacks [90].

Increased blood sugar stability may benefit migraineurs [92]. In an experiment run in 2018, a low-glycemic-index diet was able to decrease attack frequency in the first month after beginning this diet [93].

Relatively recent epigenetic diets aim to influence the mechanisms of cellular structures, e.g., mitochondria or some molecules, e.g., DNA, using specific dietary ingredients. Their name came from Hardy and Tollefsboll, who have shown the possibility of dietary ingredients influencing the epigenetic system of patients with certain disorders, and that many ingredients might have a role in preventing diseases [94]. In migraine prevention and treatment, we should focus on compounds that can inhibit certain mechanisms that have a role in migraine’s pathomechanism or can boost prevention. In this regard, one of the most promising ingredients is folate or vitamin B9, which has a role in DNA methylation. A previous study has found that abnormal mitochondrial DNA methylation occurs in migraine patients [95]; thus, folate will be a target of future studies in this field. Besides folate, riboflavin, or vitamin B2, is another compound that influences mitochondrial mechanisms. In migraine patients, riboflavin intake can inhibit the development of attacks [96,97,98,99] and their duration [100], which confirms the neuroprotective effect of riboflavin. On the other hand, besides mitochondrial DNA methylation, we should focus on histone modification in chromatin, as it can influence protein and RNA production, which can be adjusted by introducing certain dietary compounds into a patient’s diet. A histone deacetylase inhibitor drug, namely valproate, is usually an effective treatment option in epilepsy and in different types of migraine [101,102], and is considered an epigenetic drug [103]; it is another future treatment option and an alternative to epigenetic diets. In addition to this, an experimental cortical CSD model has shown that CSD can induce chromatin modification in rats [104], which proves a link between migraine and histone modification. The usage of epigenetic diets requires further research, possibly with the epigenetic profiles of patients defined prior to introduction [105].

Other much-researched diets are Dietary Approaches to Stop Hypertension (DASH), the Mediterranean Diet, and the Mediterranean-DASH Intervention for Neurodegenerative Delay (MIND) Diet, which have not been exclusively examined in the context of migraine. The DASH diet was originally developed to counter hypertension, and focuses on the consumption of fruits, vegetables, and whole grains while refraining from sodium, sweets, or saturated fats [92]. There are few data on migraine and the DASH diet, but what we do have are positive; the diet decreases the Migraine Index, which measures the frequency and severity of the attacks, it reduces the Headache Diary Result, which shows the frequency and duration of migraine pain, and also cuts back the Migraine Headache Index Score, which surveys the frequency, the duration and the severity of attacks in women [92]. The Mediterranean Diet, which focuses on vegetables, legumes, fruits, nuts, olive oil, and limited animal-based meat consumption, yields similar results. The MIND Diet was originally developed for the prevention of Alzheimer’s disease [106], and has very little effect on migraine pain in women [107]. Relatively new data have also shown that tryptophan-rich nutrition (flaxseed, salami, lentils, turkey, nuts, and eggs) in a healthy diet can decrease the odds of migraine attacks in migraineurs [79,108].

Tryptophan has a distinguished role in migraine’s pathomechanism, since it is also a precursor of serotonin and kynurenines. Migraine patients have attenuated levels of serotonin and tryptophan during interictal periods, and show enhanced levels of serotonin and tryptophan under ictal periods [109,110]. Besides serotonin, the other pathway produced by tryptophan is the kynurenine pathway. Abnormal concentrations of kynurenines have been described in chronic migraine patients [111] and in a nitroglycerin model of migraine in rats [112]; thus, tryptophan-rich diets might have a role in the prevention and treatment of migraine. However, this requires further investigation, as acute and chronic intake of tryptophan has produced contradictory results [113,114].

### 4.3. Pre- and Probiotics

Plenty of research data have shown that there is pivotal crosstalk between the brain and gut, as we have discussed earlier. The modification of gut microflora can prevent or even treat certain disorders.

Prebiotics are fermentable food ingredients that have a beneficial effect on the health of their host [115,116], and these substances serve as food for probiotics, which are usually bacteria. Usage of probiotics might be effective in neurological disorders, e.g., Parkinson’s disease [117]. Currently, there is a lack of information about the possible positive roles of pre- or probiotics and the gut–brain axis in migraine’s pathomechanism, but we have enough data to justify further research in this field. We know that enhanced intestinal permeability and pro-inflammatory items can be found in many intestinal disorders, and these conditions might influence the trigeminovascular system, thus provoking migraine attacks [118]. The fact that some other disorders, namely allergies and asthma, are connected to migraine proves the hypothesis that inflammatory processes can contribute to migraine pathomechanism [119,120]. Adequate fiber and low-glycemic-index diets generate the production of normal gut flora and have a role in migraine prevention [25], as well. Nowadays, we have only little human data on the application of pre-and probiotics in migraine patients. In an experiment, female patients took a mixture of different strains of Lactobacillus, Bifidobacterium, and Streptococcus for 12 weeks, and the frequency and the applied numbers of painkillers were reduced, but the severity and duration of attacks did not change [121]. Another study has shown that application of a mixture of Bacillus, Bifidobacterium, Lactobacillus and Streptococcus strains for 8–10 weeks can decrease the severity and frequency of attacks in episodic migraine patients, and reduce the frequency, severity, duration, and the number of drugs taken per day in chronic migraine patients [122]. In one other experiment, researchers could not detect any change in migraine frequency, and drug usage after a 12-week application of a mixture of different Bifidobacterium and Lactobacillus strains [123]. On the other hand, we should note that some Lactic acid bacteria can produce biogenic amines (e.g., histamine, tyramine), which can cause an increase or decrease in blood pressure, thus contributing to triggering headache in people [65].

**Figure 5:**
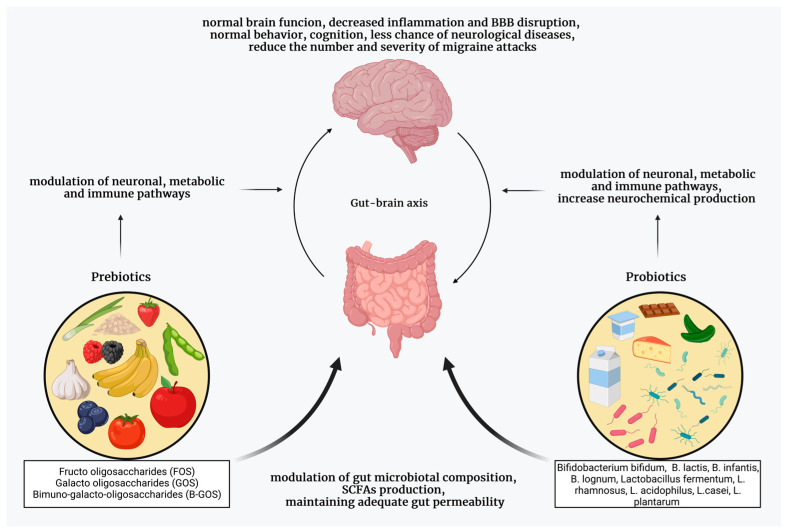
The beneficial effect of pre- and probiotics. Recently, pre-and probiotics have become the focus of migraine treatment, as the gut microbiota can influence the function of the CNS through various mechanisms. Taking pre- and probiotics can help restore and maintain a healthy gut microbiome, and can thus affect the frequency and severity of migraines. SCFAs, short-chain fatty acids, BBB, blood–brain barrier.

To summarize, we must acknowledge that we do not have enough data concerning the usage of pre- or probiotics in migraine prevention and treatment yet. Despite all of this, some bacteria, such as *Bacillus subtilis*, *Lactobacillus casei*, *Lactobacillus acidophilus*, *Lactobacillus gasseri*, *Lactobacillus bulgaricus*, *Lactobacillus helveticus*, *Lactobacillus plantarum*, *Lactobacillus rhamnosus*, *Bifidobacterium lactis*, *Bifidobacterium longum*, *Bifidobacterium breve*, *Bifidobacterium bifidum*, and *Streptococcus thermophilus* require further study, but might prove to be a good alternative treatment in the future (Figure 5).

## 5. Conclusions

Migraine is a prevalent, recurrent, and multifactorial disorder of the CNS, and it seems gut microbiota dysbiosis contributes to this disease; thus, preserving the species richness and composition of the microbiome and improving the stability of the micro-ecosystem can improve the quality of life of migraine patients, and reduce the risk of migraine headaches and the various GI diseases comorbid with migraine.

Taken together, we can summarize that there are many possibilities for migraine treatment and prevention in the context of nutrition, and combinations of these diets might serve as good additional therapy in migraine management. In addition to this, it is important to note that patients should consult a dietician in this regard. Generally, we can recommend that patients with migraine should pay attention to meal regularity and weight loss, because healthier diets and a normal body mass index (BMI) can decrease the probability of migraine.

## Data Availability

Not applicable.

## Abbreviations

5-HT	serotonin
Ach	acetylcholine
BMI	body mass index
CD	celiac disease
CGRP	calcitonin gene-related peptide
CNS	central nervous system
CRH	corticotrophin-releasing hormone
CTX	cortex
CSD	cortical spreading depression
DASH	Dietary Approaches to Stop Hypertension
DMV	dorsal motor nucleus of the vagus
eNOS	endothelial nitric oxide synthase
GABA	gamma-aminobutyric acid
GI	gastrointestinal
hTh	hypothalamus
IBD	inflammatory bowel disease
IBS	irritable bowel syndrome
IL	interleukin
LC	locus coeruleus
LP	lateral posterior nucleus
LPS	lipopolysaccharides
MIND	Mediterranean-DASH Intervention for Neurodegenerative Delay
NKA	neurokinin A
NO	nitric-oxide
NPY	neuropeptide Y
PACAP	pituitary adenylate cyclase-activating polypeptide
PAG	periaqueductal grey matter
PN	personalized or precision nutrition
SCFAs	short-chain fatty acids
SP	substance P
SPG	sphenopalatine ganglion
SpV	spinal trigeminal nucleus caudalis
SSN	superior salivatory nucleus
TCC	trigeminocervical complex
TG	trigeminal ganglion
Th	thalamus
TNC	trigeminal nucleus caudalis
TNF-α	tumor necrosis factor-α
V1	ophthalmic nerve
V2	maxillary nerve
V3	mandibular nerve
VIP	vasoactive intestinal peptide
VPL	ventral posterolateral nucleus
VPM	ventral posteromedial nucleus

## References

[1] Lipton R.B., Bigal M.E., Steiner T.J., Silberstein S.D., Olesen J. (2004). Classification of primary headaches. Neurology.

[2] Headache Classification Committee of the International Headache Society (IHS) (2013). The International Classification of Headache Disorders, 3rd edition, (beta version). Cephalalgia.

[3] GBD 2016 Disease and Injury Incidence and Prevalence Collaborators (2017). Global, regional, and national incidence, prevalence, and years lived with disability for 328 diseases and injuries for 195 countries, 1990–2016: A systematic analysis for the Global Burden of Disease Study 2016. Lancet.

[4] Al Ghadeer H.A., AlSalman S.A., Albaqshi F.M., Alsuliman S.R., Alsowailem F.A., Albusror H.A., AlAbdi Z.I., Alwabari E.M., Alturaifi Z.A., AlHajji A.M. (2021). Quality of Life and Disability Among Migraine Patients: A Single-Center Study in AlAhsa, Saudi Arabia. Cureus.

[5] Goadsby P.J., Holland P.R., Martins-Oliveira M., Hoffmann J., Schankin C., Akerman S. (2017). Pathophysiology of Migraine: A Disorder of Sensory Processing. Physiol. Rev..

[6] Schulte L.H., May A. (2016). The migraine generator revisited: Continuous scanning of the migraine cycle over 30 days and three spontaneous attacks. Brain.

[7] Karsan N., Bose P., Goadsby P.J. (2018). The Migraine Premonitory Phase. Contin. Lifelong Learn. Neurol..

[8] Lai J., Dilli E. (2020). Migraine Aura: Updates in Pathophysiology and Management. Curr. Neurol. Neurosci. Rep..

[9] Chen P.K., Wang S.J. (2018). Non-headache symptoms in migraine patients. F1000Research.

[10] Qubty W., Patniyot I. (2020). Migraine Pathophysiology. Pediatr. Neurol..

[11] Moskowitz M.A., Reinhard J.F., Romero J., Melamed E., Pettibone D.J. (1979). Neurotransmitters and the fifth cranial nerve: Is there a relation to the headache phase of migraine?. Lancet.

[12] Zhang X., Levy D., Kainz V., Noseda R., Jakubowski M., Burstein R. (2011). Activation of central trigeminovascular neurons by cortical spreading depression. Ann. Neurol..

[13] Bernstein C., Burstein R. (2012). Sensitization of the trigeminovascular pathway: Perspective and implications to migraine pathophysiology. J. Clin. Neurol..

[14] Spekker E., Tanaka M., Szabó Á., Vécsei L. (2021). Neurogenic Inflammation: The Participant in Migraine and Recent Advancements in Translational Research. Biomedicines.

[15] Borkum J.M. (2021). Brain Energy Deficit as a Source of Oxidative Stress in Migraine: A Molecular Basis for Migraine Susceptibility. Neurochem. Res..

[16] Edvinsson L. (2019). Role of CGRP in Migraine. Handb. Exp. Pharmacol..

[17] Robblee J., Starling A.J. (2019). SEEDS for success: Lifestyle management in migraine. Cleve Clin. J. Med..

[18] Gazerani P. (2020). Migraine and Diet. Nutrients.

[19] Peroutka S.J. (2014). What turns on a migraine? A systematic review of migraine precipitating factors. Curr. Pain Headache Rep..

[20] Nowaczewska M., Wiciński M., Kaźmierczak W., Kaźmierczak H. (2020). To Eat or Not to Eat: A Review of the Relationship between Chocolate and Migraines. Nutrients.

[21] Theoharides T.C., Donelan J., Kandere-Grzybowska K., Konstantinidou A. (2005). The role of mast cells in migraine pathophysiology. Brain Res. Rev..

[22] Ramachandran R. (2018). Neurogenic inflammation and its role in migraine. Semin. Immunopathol..

[23] Holzer P., Farzi A. (2014). Neuropeptides and the microbiota-gut-brain axis. Adv. Exp. Med. Biol..

[24] Kohler D.R., Goldspiel B.R. (1991). Ondansetron: A serotonin receptor (5-HT3) antagonist for antineoplastic chemotherapy-induced nausea and vomiting. DICP.

[25] Arzani M., Jahromi S.R., Ghorbani Z., Vahabizad F., Martelletti P., Ghaemi A., Sacco S., Togha M., School of Advanced Studies of the European Headache Federation (EHF-SAS) (2020). Gut-brain Axis and migraine headache: A comprehensive review. J. Headache Pain.

[26] Cámara-Lemarroy C.R., Rodriguez-Gutierrez R., Monreal-Robles R., Marfil-Rivera A. (2016). Gastrointestinal disorders associated with migraine: A comprehensive review. World J. Gastroenterol..

[27] Hindiyeh N.A., Zhang N., Farrar M., Banerjee P., Lombard L., Aurora S.K. (2020). The Role of Diet and Nutrition in Migraine Triggers and Treatment: A Systematic Literature Review. Headache.

[28] Thursby E., Juge N. (2017). Introduction to the human gut microbiota. Biochem. J..

[29] Rowland I., Gibson G., Heinken A., Scott K., Swann J., Thiele I., Tuohy K. (2018). Gut microbiota functions: Metabolism of nutrients and other food components. Eur. J. Nutr..

[30] Lin L., Zhang J. (2017). Role of intestinal microbiota and metabolites on gut homeostasis and human diseases. BMC Immunol..

[31] Romijn J.A., Corssmit E.P., Havekes L.M., Pijl H. (2008). Gut-brain axis. Curr. Opin. Clin. Nutr. Metab. Care.

[32] Wang Y., Kasper L.H. (2014). The role of microbiome in central nervous system disorders. Brain Behav. Immun..

[33] Zang Y., Lai X., Li C., Ding D., Wang Y., Zhu Y. (2023). The Role of Gut Microbiota in Various Neurological and Psychiatric Disorders-An Evidence Mapping Based on Quantified Evidence. Mediat. Inflamm..

[34] Mayer E.A., Tillisch K., Gupta A. (2015). Gut/brain axis and the microbiota. J. Clin. Investig..

[35] Shreiner A.B., Kao J.Y., Young V.B. (2015). The gut microbiome in health and in disease. Curr. Opin. Gastroenterol..

[36] Gomaa E.Z. (2020). Human gut microbiota/microbiome in health and diseases: A review. Antonie Van Leeuwenhoek.

[37] Ustianowska K., Ustianowski Ł., Machaj F., Gorący A., Rosik J., Szostak B., Szostak J., Pawlik A. (2022). The Role of the Human Microbiome in the Pathogenesis of Pain. Int. J. Mol. Sci..

[38] Amaral F.A., Sachs D., Costa V.V., Fagundes C.T., Cisalpino D., Cunha T.M., Ferreira S.H., Cunha F.Q., Silva T.A., Nicoli J.R. (2008). Commensal microbiota is fundamental for the development of inflammatory pain. Proc. Natl. Acad. Sci. USA.

[39] Strandwitz P., Kim K.H., Terekhova D., Liu J.K., Sharma A., Levering J., McDonald D., Dietrich D., Ramadhar T.R., Lekbua A. (2019). GABA-modulating bacteria of the human gut microbiota. Nat. Microbiol..

[40] Cryan J.F., O’Riordan K.J., Sandhu K., Peterson V., Dinan T.G. (2020). The gut microbiome in neurological disorders. Lancet Neurol..

[41] Baj A., Moro E., Bistoletti M., Orlandi V., Crema F., Giaroni C. (2019). Glutamatergic Signaling Along the Microbiota-Gut-Brain Axis. Int. J. Mol. Sci..

[42] Hoffmann J., Charles A. (2018). Glutamate and Its Receptors as Therapeutic Targets for Migraine. Neurotherapeutics.

[43] Gonzalez A., Hyde E., Sangwan N., Gilbert J.A., Viirre E., Knight R. (2016). Migraines Are Correlated with Higher Levels of Nitrate-, Nitrite-, and Nitric Oxide-Reducing Oral Microbes in the American Gut Project Cohort. mSystems.

[44] Chen J., Wang Q., Wang A., Lin Z. (2020). Structural and Functional Characterization of the Gut Microbiota in Elderly Women with Migraine. Front. Cell. Infect. Microbiol..

[45] Aamodt A.H., Stovner L.J., Hagen K., Zwart J.A. (2008). Comorbidity of headache and gastrointestinal complaints. The Head-HUNT Study. Cephalalgia.

[46] Aurora S.K., Shrewsbury S.B., Ray S., Hindiyeh N., Nguyen L. (2021). A link between gastrointestinal disorders and migraine: Insights into the gut-brain connection. Headache.

[47] Schuppan D., Junker Y., Barisani D. (2009). Celiac disease: From pathogenesis to novel therapies. Gastroenterology.

[48] Van Hemert S., Breedveld A.C., Rovers J.M., Vermeiden J.P., Witteman B.J., Smits M.G., de Roos N.M. (2014). Migraine associated with gastrointestinal disorders: Review of the literature and clinical implications. Front. Neurol..

[49] Chang F.Y., Lu C.L. (2013). Irritable bowel syndrome and migraine: Bystanders or partners?. J. Neurogastroenterol. Motil..

[50] Dimitrova A.K., Ungaro R.C., Lebwohl B., Lewis S.K., Tennyson C.A., Green M.W., Babyatsky M.W., Green P.H. (2013). Prevalence of migraine in patients with celiac disease and inflammatory bowel disease. Headache.

[51] Crawford J., Liu S., Tao F. (2022). Gut microbiota and migraine. Neurobiol. Pain.

[52] Li C., Yu S., Li H., Zhou J., Liu J., Tang W., Zhang L. (2017). Clinical features and risk factors for irritable bowel syndrome in Migraine patients. Pak. J. Med. Sci..

[53] Boyle R., Behan P.O., Sutton J.A. (1990). A correlation between severity of migraine and delayed gastric emptying measured by an epigastric impedance method. Br. J. Clin. Pharmacol..

[54] Waelkens J. (1984). Dopamine blockade with domperidone: Bridge between prophylactic and abortive treatment of migraine? A dose-finding study. Cephalalgia.

[55] Acosta A., Camilleri M. (2015). Prokinetics in gastroparesis. Gastroenterol. Clin. N. Am..

[56] Friedman B.W., Mulvey L., Esses D., Solorzano C., Paternoster J., Lipton R.B., Gallagher E.J. (2011). Metoclopramide for acute migraine: A dose-finding randomized clinical trial. Ann. Emerg. Med..

[57] Shakhatreh M., Jehangir A., Malik Z., Parkman H.P. (2019). Metoclopramide for the treatment of diabetic gastroparesis. Expert. Rev. Gastroenterol. Hepatol..

[58] Kopishinskaya S.V., Gustov A.V. (2015). Gluten migraine. Zhurnal Nevrologii Psikhiatrii Imeni SS Korsakova.

[59] Zis P., Julian T., Hadjivassiliou M. (2018). Headache Associated with Coeliac Disease: A Systematic Review and Meta-Analysis. Nutrients.

[60] Mormile R. (2014). Celiac disease and migraine: Is there a common backstage?. Int. J. Color. Dis..

[61] Voruganti V.S. (2023). Precision Nutrition: Recent Advances in Obesity. Physiology.

[62] Muniesa G., Martinez J.A., González-Muniesa P. (2019). Precision Nutrition and Metabolic Syndrome Management. Nutrients.

[63] Kokavec A. (2016). Migraine: A disorder of metabolism?. Med. Hypotheses.

[64] Rainero I., Govone F., Gai A., Vacca A., Rubino E. (2018). Is Migraine Primarily a Metaboloendocrine Disorder?. Curr. Pain Headache Rep..

[65] Martin V.T., Vij B. (2016). Diet and Headache: Part 1. Headache J. Head Face Pain.

[66] Cairns B.E. (2016). Influence of pro-algesic foods on chronic pain conditions. Expert. Rev. Neurother..

[67] Scopp A.L. (1991). MSG and hydrolyzed vegetable protein induced headache: Review and case studies. Headache.

[68] Shimada A., Cairns B.E., Vad N., Ulriksen K., Pedersen Lynge A.M., Svensson P., Baad-Hansen L. (2013). Headache and mechanical sensitization of human pericranial muscles after repeated intake of monosodium glutamate (MSG). J. Headache Pain.

[69] Juliano L.M., Griffiths R.R. (2004). A critical review of caffeine withdrawal: Empirical validation of symptoms and signs, incidence, severity, and associated features. Psychopharmacology.

[70] Nehlig A. (2016). Effects of coffee/caffeine on brain health and disease: What should I tell my patients?. Pract. Neurol..

[71] Shapiro R.E. (2008). Caffeine and headaches. Curr. Pain Headache Rep..

[72] Boardman H.F., Thomas E., Millson D.S., Croft P.R. (2005). Psychological, sleep, lifestyle, and comorbid associations with headache. Headache.

[73] Zhu H., Xing Y., Akan O.D., Yang T. (2023). Alcohol-Induced Headache with Neuroinflammation: Recent Progress. Fermentation.

[74] Panconesi A. (2008). Alcohol and migraine: Trigger factor, consumption, mechanisms. A review. J. Headache Pain.

[75] Lippi G., Mattiuzzi C., Cervellin G. (2014). Chocolate and migraine: The history of an ambiguous association. Acta Biomed..

[76] Ellam S., Williamson G. (2013). Cocoa and human health. Annu. Rev. Nutr..

[77] Cady R.J., Durham P.L. (2010). Cocoa-enriched diets enhance expression of phosphatases and decrease expression of inflammatory molecules in trigeminal ganglion neurons. Brain Res..

[78] Magrone T., Russo M.A., Jirillo E. (2017). Cocoa and Dark Chocolate Polyphenols: From Biology to Clinical Applications. Front. Immunol..

[79] Razeghi Jahromi S., Togha M., Ghorbani Z., Hekmatdoost A., Khorsha F., Rafiee P., Shirani P., Nourmohammadi M., Ansari H. (2019). The association between dietary tryptophan intake and migraine. Neurol. Sci..

[80] Abbey M.J., Patil V.V., Vause C.V., Durham P.L. (2008). Repression of calcitonin gene-related peptide expression in trigeminal neurons by a Theobroma cacao extract. J. Ethnopharmacol..

[81] Nattagh-Eshtivani E., Sani M.A., Dahri M., Ghalichi F., Ghavami A., Arjang P., Tarighat-Esfanjani A. (2018). The role of nutrients in the pathogenesis and treatment of migraine headaches: Review. Biomed. Pharmacother..

[82] Sun-Edelstein C., Mauskop A. (2009). Foods and supplements in the management of migraine headaches. Clin. J. Pain.

[83] Martin V.T., Behbehani M.M. (2001). Toward a rational understanding of migraine trigger factors. Med. Clin. N. Am..

[84] Council of Scientific Affairs (1985). Aspartame: Review of safety issues. JAMA.

[85] Van den Eeden S.K., Koepsell T.D., Longstreth W.T., van Belle G., Daling J.R., McKnight B. (1994). Aspartame ingestion and headaches: A randomized crossover trial. Neurology.

[86] Loehler S.M., Glaros A. (1988). The effect of aspartame on migraine headache. Headache.

[87] Ozon A.O., Karadas O., Ozge A. (2018). Efficacy of diet restriction on migraines. Arch. Neuropsychiatry.

[88] Aydinla E.I., Dikmen P.Y., Tiftikci A., Saruc M., Aksu M., Gunsoy H.G., Tozun N. (2013). IgG-based elimination diet in migraine plus irritable bowel syndrome. Headache.

[89] Jahromi S.R., Ghorbani Z., Martelletti P., Lampl C., Togha M., On behalf of the School of Advanced Studies of the European Headache Federation (EHF-SAS) (2019). Association of diet and headache. J. Headache Pain.

[90] Gross E.C., Klement R.J., Schoenen J., D’Agostino D.P., Fischer D. (2019). Potential Protective Mechanisms of Ketone Bodies in Migraine Prevention. Nutrients.

[91] Di Lorenzo C., Coppola G., Bracaglia M., Di Lenola D., Evangelista M., Sirianni G., Rossi P., Di Lorenzo G., Serrao M., Parisi V. (2016). Cortical functional correlates of responsiveness to short-lasting preventive intervention with ketogenic diet in migraine: A multimodal evoked potentials study. J. Headache Pain.

[92] Moskatel L.S., Zhang N. (2022). Migraine and Diet: Updates in Understanding. Curr. Neurol. Neurosci. Rep..

[93] Evcili G., Utku U., Ogun M.N., Ozdemir G. (2018). Early and long period follow-up results of low glycemic index diet for migraine prophylaxis. J. Turk. Soc. Algol..

[94] Hardy T.M., Tollefsbol T.O. (2011). Epigenetic diet: Impact on the epigenome and cancer. Epigenomics.

[95] Fila M., Pawłowska E., Blasiak J. (2019). Mitochondria in migraine pathophysiology—Does epigenetics play a role?. Arch. Med. Sci..

[96] Thompson D.F., Saluja H.S. (2017). Prophylaxis of migraine headaches with riboflavin: A systematic review. J. Clin. Pharm. Ther..

[97] Daniel O., Mauskop A. (2016). Nutraceuticals in acute and prophylactic treatment of migraine. Curr. Treat. Options Neurol..

[98] Marashly E.T., Bohlega S.A. (2017). Riboflavin has neuroprotective potential: Focus on Parkinson’s disease and migraine. Front. Neurol..

[99] Namazi N., Heshmati J., Tarighat-Esfanjani A. (2015). Supplementation with riboflavin (Vitamin B2) for migraine prophylaxis in adults and children: A review. Int. J. Vitam. Nutr. Res..

[100] Schoenen J., Jacquy J., Lenaerts M. (1998). Effectiveness of high-dose riboflavin in migraine prophylaxis. A randomized controlled trial. Neurology.

[101] Karimi N., Tavakoli M., Charati J.Y., Shamsizade M. (2017). Single-dose intravenous sodium valproate (Depakine) versus dexamethasone for the treatment of acute migraine headache: A double-blind randomized clinical trial. Clin. Exp. Emerg. Med..

[102] Liu F., Ma T., Che X., Wang Q., Yu S. (2017). The efficacy of venlafaxine, flunarizine, and valproic acid in the prophylaxis of vestibular migraine. Front. Neurol..

[103] Ganesan A., Arimondo P.B., Rots M.G., Jeronimo C., Berdasco M. (2019). The timeline of epigenetic drug discovery: From reality to dreams. Clin. Epigenet..

[104] Passaro D., Rana G., Piscopo M., Viggiano E., De Luca B., Fucci L. (2010). Epigenetic chromatin modifications in the cortical spreading depression. Brain Res..

[105] Gazerani P. (2018). Current Evidence on the Role of Epigenetic Mechanisms in Migraine: The Way Forward to Precision Medicine. OBM Genet..

[106] Morris M.C., Tangney C.C., Wang Y., Sacks F.M., Barnes L.L., Bennett D.A., Aggarwal N.T. (2015). MIND diet slows cognitive decline with aging. Alzheimers Dement..

[107] Askarpour M., Yarizadeh H., Sheikhi A., Khorsha F., Mirzaei K. (2020). Associations between adherence to MIND diet and severity, duration and frequency of migraine headaches among migraine patients. BMC Res. Notes.

[108] Escott-Stump S. (2011). Nutrition and Diagnosis-Related Care.

[109] Deen M., Christensen C.E., Hougaard A., Hansen H.D., Knudsen G.M., Ashina M. (2017). Serotonergic mechanisms in the migraine brain—A systematic review. Cephalalgia.

[110] Gasparini C.F., Smith R.A., Griffiths L.R. (2017). Genetic and biochemical changes of the serotonergic system in migraine pathobiology. J. Headache Pain.

[111] Curto M., Lionetto L., Fazio F., Mitsikostas D.D., Martelletti P. (2015). Fathoming the kynurenine pathway in migraine: Why understanding the enzymatic cascades is still critically important. Intern. Emerg. Med..

[112] Nagy-Grócz G., Laborc K.F., Veres G., Bajtai A., Bohár Z., Zádori D., Fejes-Szabó A., Spekker E., Vécsei L., Párdutz Á. (2017). The Effect of Systemic Nitroglycerin Administration on the Kynurenine Pathway in the Rat. Front. Neurol..

[113] Sicuteri F. (1973). The ingestion of serotonin precursors (L-5-hydroxytryptophan and L-tryptophan) improves migraine headache. Headache.

[114] Gedye A. (2001). Hypothesized treatment for migraines using low doses of tryptophan, niacin, calcium, caffeine, and acetylsalicylic acid. Med. Hypotheses.

[115] Roberfroid M. (2007). Prebiotics: The concept revisited. J. Nutr..

[116] Gibson G.R., Hutkins R., Sanders M.E., Prescott S.L., Reimer R.A., Salminen S.J., Scott K., Stanton C., Swanson K.S., Cani P.D. (2017). Expert consensus document: The International Scientific Association for Probiotics and Prebiotics (ISAPP) consensus statement on the definition and scope of prebiotics. Nat. Rev. Gastroenterol. Hepatol..

[117] Gazerani P. (2019). Probiotics for Parkinson’s Disease. Int. J. Mol. Sci..

[118] Galland L. (2014). The Gut Microbiome and the Brain. J. Med. Food.

[119] Saberi A., Nemati S., Shakib R.J., Kazemnejad E., Maleki M. (2012). Association between allergic rhinitis and migraine. J. Res. Med. Sci..

[120] Kim S.Y., Min C., Oh D.J., Lim J.-S., Choi H.-G. (2019). Bidirectional association between asthma and migraines in adults: Two longitudinal follow-up studies. Sci. Rep..

[121] Ghavami A., Khorvash F., Heidari Z., Khalesi S., Askari G. (2021). Effect of synbiotic supplementation on migraine characteristics and inflammatory biomarkers in women with migraine: Results of a randomized controlled trial. Pharmacol. Res..

[122] Martami F., Togha M., Seifishahpar M., Ghorbani Z., Ansari H., Karimi T., Jahromi S.R. (2019). The effects of a multispecies probiotic supplement on inflammatory markers and episodic and chronic migraine characteristics: A randomized double-blind controlled trial. Cephalalgia.

[123] De Roos N.M., van Hemert S., Rovers J.M.P., Smits M.G., Witteman B.J.M. (2017). The effects of a multispecies probiotic on migraine and markers of intestinal permeability—Results of a randomized placebo-controlled study. Eur. J. Clin. Nutr..

